# A Child With Iron‐Refractory Iron Deficiency Anemia: A Rare Case Associated With Hiatal Hernia

**DOI:** 10.1002/ccr3.72223

**Published:** 2026-03-06

**Authors:** Worku Ketema, Kefyalew Taye Garie, Enatnesh Terefe Mame, Mekdes Shifeta, Wondimagegn Gizaw, Shifte Hamid, Rihana Kassim Ahmed, Tsegaye Yasin, Solomon Kelemu Leykun, Anteneh Dejene, Agete Tadewos Hirigo, Mulugeta Sitot Shibeshi

**Affiliations:** ^1^ Department of Pediatrics and Child Health Hawassa University Hawassa Ethiopia; ^2^ Department of Surgery Hawassa University Hawassa Ethiopia; ^3^ Department of Radiology Panacea Primary Hospital Hawassa Ethiopia; ^4^ School of Medical Laboratory Science Hawassa University Hawassa Ethiopia

**Keywords:** child, hiatal hernia, iron deficiency anaemia, iron‐refractory iron deficiency anaemia

## Abstract

Iron deficiency anemia remains a significant health problem in children. Gastrointestinal blood loss is a recognized cause of iron deficiency in this population. Although rare, hiatal hernia has been reported as a source of chronic gastrointestinal bleeding, potentially leading to iron refractory iron deficiency anemia (IDA). A 4‐year‐old male child with iron refractory IDA had previously received multiple blood transfusions for recurrent anemia. During evaluation for complicated pneumonia, a chest computed tomography scan incidentally revealed a hiatal hernia. Following surgical repair of the hernia, iron refractory IDA responded to iron supplementation and follow‐up complete blood counts at 2 and 6 months demonstrated normalization of hemoglobin levels. Clinicians should be aware that hiatal hernia might represent a rare but significant cause of anemia in children with iron refractory IDA.

## Key Clinical Messages

- Hiatal hernia, though rare, can cause chronic gastrointestinal bleeding and iron‐refractory iron deficiency anemia.
- Persistent or unexplained anemia should prompt evaluation for underlying structural causes.
- Early recognition and surgical repair can restore response to iron therapy, normalize hemoglobin, reduce transfusions and improve long‐term outcomes.

## 1 Introduction

Iron deficiency is the leading cause of anemia in children and is associated with significant adverse outcomes, including impaired growth and development. Globally, iron deficiency anemia (IDA) remains a major public health problem, affecting over 1.2 billion people in 2016 [1]. According to the World Health Organization, 42.6% of children under five years of age were estimated to have IDA in 2011 [2].

The causes of IDA in children include: (1) inadequate iron intake due to low dietary iron content or impaired nutritional absorption and (2) blood loss, particularly from the gastrointestinal tract (GIT) [1]. Chronic IDA resulting from occult bleeding may be associated with GIT lesions such as reflux esophagitis, hiatal hernia, peptic ulcer, polyp, hookworm infection, Meckel diverticulum or inflammatory bowel disease [1, 3].

Hiatal hernia in children may be congenital or acquired. It is often asymptomatic but can present with reflux‐related symptoms such as vomiting, aspiration, recurrent pneumonia, feeding difficulties, failure to thrive, IDA, and rarely gastric volvulus [4, 5, 6]. Hiatal hernia has also been reported as a cause of iron‐refractory anemia, defined as the absence of a hematologic response (i.e., < 1 g/dL increase in hemoglobin after 4–6 weeks of oral iron therapy) [7]. We report a case of a 4‐year‐old Ethiopian child with severe iron‐refractory iron deficiency anemia (IDA) secondary to hiatal hernia. Following surgical repair of the hernia, the IDA responded to iron therapy and follow‐up complete blood counts (CBC) at 2 and 6 months revealed a normal value of hemoglobin levels.

## 2 Case History

A 4‐year‐old male child presented with a history of loss of appetite, easy fatigability and pica since the age of 2 years. He was initially seen at a private clinic where IDA was diagnosed, although the underlying cause was not identified. The child was treated with ferrous sulphate for several months, dewormed and parents were advised to reduce cow's milk intake and provide an iron‐rich diet. Despite this management, the IDA persisted.

The child exhibited optimal growth and development and had no weight loss, night sweats, oedema, abdominal pain, vomiting, diarrhea, constipation, hematemesis, melena, haematochezia, recurrent epistaxis or bleeding from any other site. There was no history of atopy.

## 3 Investigations, Differential Diagnoses and Treatment

At his presentation to private clinic, a complete blood count (CBC) revealed severe IDA (hemoglobin (HGB) 5 g/dL, hematocrit (HCT) 16.7%, mean corpuscular volume (MCV) 65.8 femtoliter (fL), mean corpuscular hemoglobin (MCH) 18.8 pictogram (pg) and red cell distribution width (RDW) 28.9%). The patient was subsequently referred to our hospital for blood transfusion and further evaluation.

At presentation to our hospital, the child was tachycardic with pallor of the palms and soles. Anthropometric measurements were normal and there was no lymphadenopathy, hepatosplenomegaly, ascites, bone tenderness or petechial rash. He received a blood transfusion and underwent evaluation for the underlying cause of IDA. Laboratory studies showed an elevated reticulocyte percentage (4.57%) and absolute reticulocyte count (0.126 × 10^6^/μL) with increased total iron‐binding capacity (378 μg/dL). Serum iron (15 μg/dL) and transferrin saturation (3.97%) were markedly low, confirming iron deficiency. However, the serum ferritin level was normal (27.4 ng/mL), which may be attributed to the recent blood transfusion. A peripheral blood film was prepared and stained to assess for hemoparasites. The smear was negative for malaria and other hemoparasites and the erythrocyte sedimentation rate was within normal limits (20 mm/h). The child was previously referred to Black Lion Hospital, Addis Ababa, where intravenous iron sucrose therapy was initiated for iron‐deficiency anemia. Iron sucrose was administered at 6 mg/kg, with second and third doses scheduled during subsequent visits to correct anemia and reduce the need for further blood transfusions. The *Helicobacter pylori* ( *H. pylori* ) stool antigen test was positive and the child received triple eradication therapy, with subsequent testing confirming clearance. Stool examination was positive for occult blood, with no ova or parasites detected. Endoscopic evaluation was planned; however, pediatric endoscopy was not available for this age at our center and the parents were advised to seek care at a facility where the service is accessible. Abdominal ultrasound and echocardiography were unremarkable. Lactate dehydrogenase, renal and liver function tests were within normal limits. During 2 years of follow‐up at our hospital, the child required multiple blood transfusions, with hemoglobin levels ranging from 4.3 to 9.2 g/dL.

**FIGURE 1:**
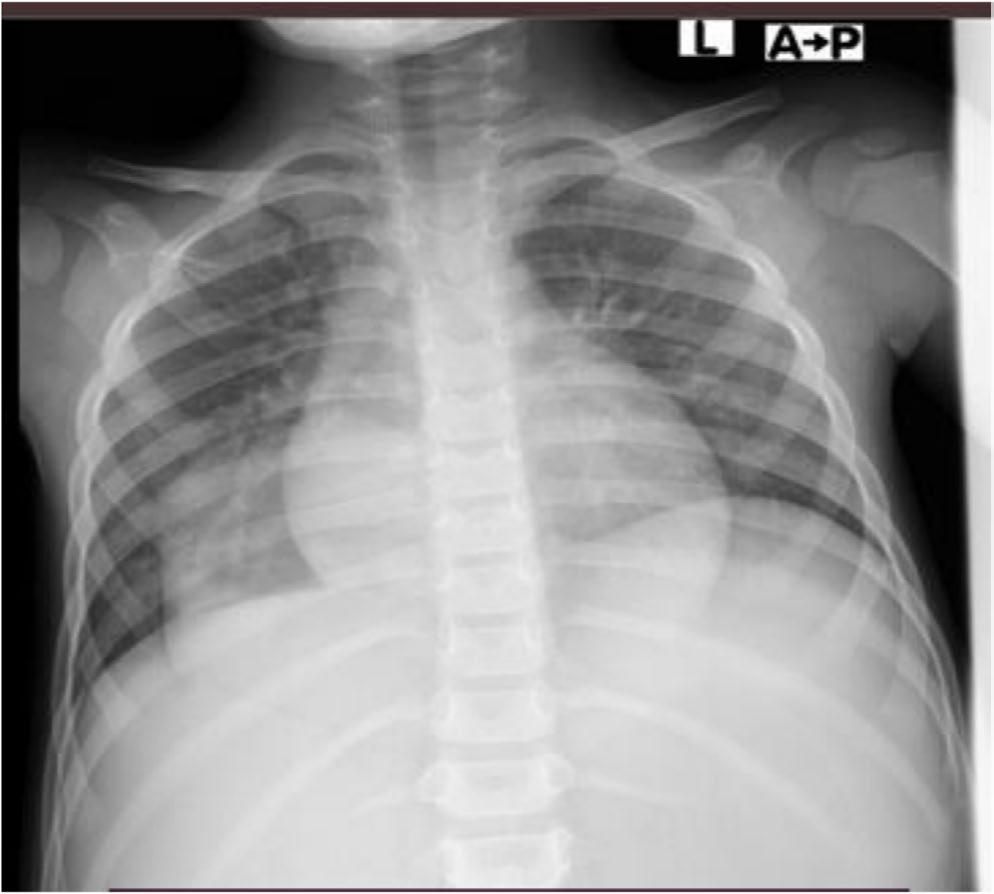
Chest x‐ray of the child showing a right lower lung zone well‐defined opacity.

**FIGURE 2:**
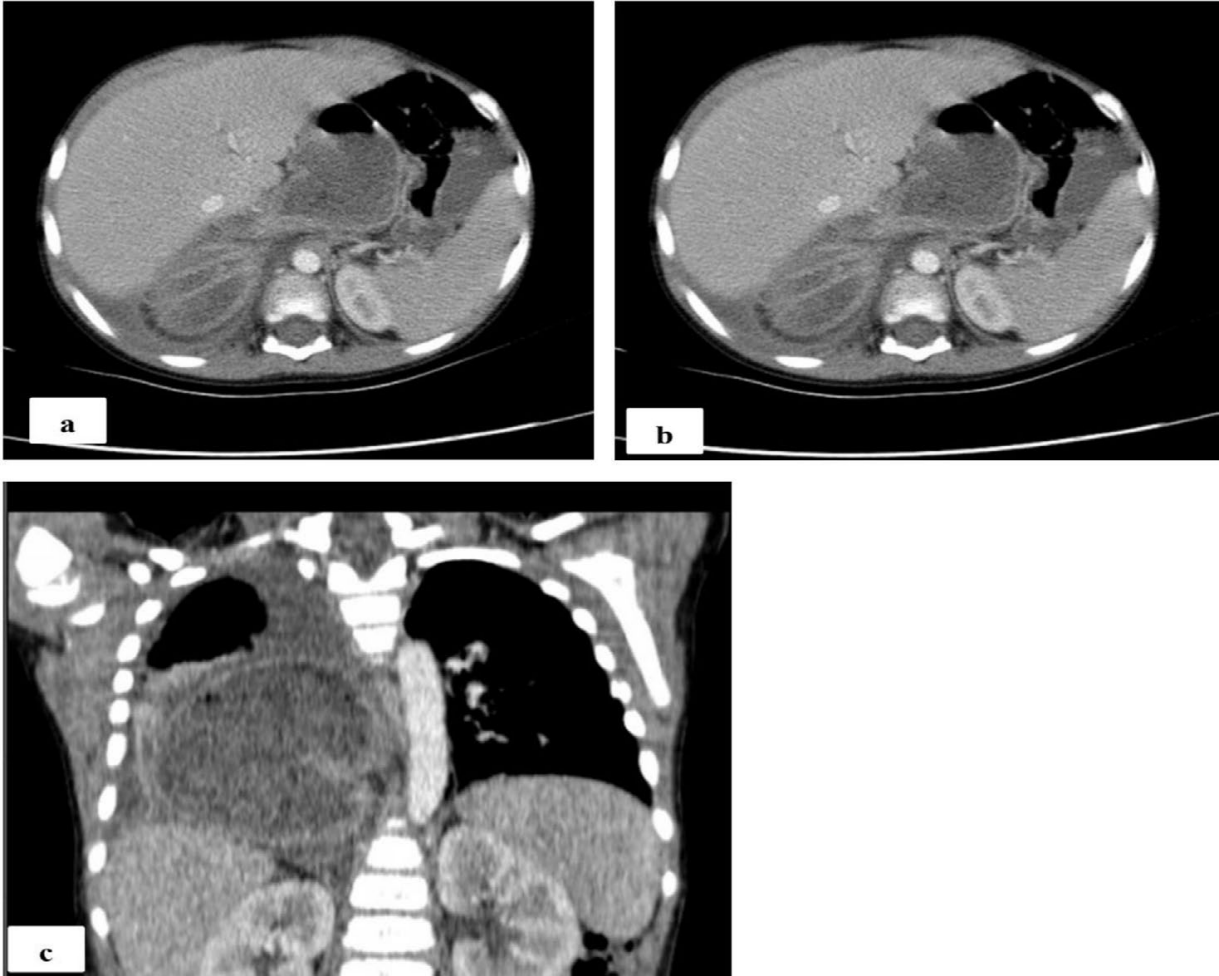
CT scan findings. (a) Contrast enhanced axial chest CT showing large trans‐hiatial herniation of the proximal stomach to the level of the gastric mid body, herniated stomach occupying the lower posterior mediastinum and the right lower thoracic cavity. (b) Contrast enhanced chest CT showing large trans‐hiatial herniation of the proximal stomach to the level of the gastric mid body with incomplete gastric volvulus. (c) Coronal reconstruction of contrast enhanced chest CT showing the herniated stomach occupying the lower posterior mediastinum and the right lower thoracic cavity.

Although awaiting referral for endoscopic evaluation, he developed severe pneumonia (fast breathing, cough, tachypnea, intercostal and subcostal retraction with decreased air entry over right lower lobe). Chest X‐ray revealed a well‐defined opacity in the right lower lung zone (Figure 1), prompting a chest computed tomography (CT) scan. The CT scan demonstrated a large trans‐hiatal herniation of the proximal stomach up to the mid‐body, associated with incomplete gastric volvulus. The herniated stomach occupied the lower posterior mediastinum and right lower thoracic cavity (Figure 2).

## 4 Conclusion and Results

A diagnosis of iron refractory IDA secondary to chronic gastrointestinal blood loss from hiatal hernia was ultimately established. After resolution of the pneumonia, the patient underwent surgical repair of the hiatal hernia. Intraoperatively, the cardia and fundus of the stomach were found herniated through a defect on the right side of the esophagus into the thoracic cavity, enclosed with a sack. The herniated content was reduced, the sack excised, and the defect in the diaphragm crus was approximated with interrupted sutures at four sites. The distal 3 cm of the esophagus was positioned within the abdominal cavity.

The postoperative course was uneventful and the patient was discharged on the fifth day with pantoprazole and ferrous sulfate supplementation. At discharge, hisHGB was 8.1 g/dL and HCT was 23.6%. He attended three follow‐up visits at the specialty referral clinic. The first visit included an abdominal ultrasound and CBC, which were within normal limits (WBC 6.85 × 10^3^/μL, HGB 13 g/dL, HCT 36.9%, platelets 356 × 10^3^/μL). At the second visit, pantoprazole therapy was continued, with the next follow‐up scheduled after two months. Subsequent evaluations demonstrated complete resolution of iron deficiency anemia, with normal hemoglobin levels of 14 and 14.7 g/dL at 2 and 6 months postsurgery, respectively.

## 5 Discussion

We report an incidental paraesophageal hiatal hernia in a 4‐year‐old Ethiopian child with severe, chronic IDA, identified during chest X‐ray and CT scan performed for complicated pneumonia. While iron refractory IDA is most often acquired and related to gastrointestinal (GI) bleeding, a rare genetic form has also been described [7]. In this patient, a positive fecal occult blood test suggested a GI source of bleeding. Chronic occult GI blood loss in children may result from pathologic conditions such as reflux esophagitis, gastritis, peptic ulcer disease, hookworm infection, colitis, eosinophilic gastroenteritis, celiac disease, inflammatory bowel disease, polyposis, Meckel's diverticulum, or vascular malformations [8]. Although endoscopic evaluation was not performed, the hiatal hernia was considered the most likely cause of the iron refractory IDA, as anemia resolved with oral iron supplementation following surgical repair. In contrast, eradication therapy for *H. pylori* and repeated deworming had failed to improve the anemia, making helminthic infection and/or *H. pylori* unlikely contributors. Furthermore, the absence of clinical or laboratory evidence of inflammatory bowel disease, colitis, celiac disease, or eosinophilic gastroenteritis supported exclusion of these conditions as potential sources of bleeding in this child.

Hiatal hernia is uncommon in children and is often asymptomatic. When symptomatic, it may present with anemia, recurrent respiratory infections, failure to thrive, dysphagia, early satiety, or vomiting [9]. Several studies have reported that hiatal hernia can cause chronic GI bleeding leading to iron refractory IDA, which typically resolves after surgical repair [4, 10, 11, 12]. Proposed mechanisms for the development of IDA in patients with hiatal hernia include hemorrhage from the area of the stomach which rides over the crus at the neck of the hiatal sac [13], bleeding from Cameron lesions (linear gastric ulcers within the hernia) [14], and hernia‐associated esophagitis [15]. However, in our patient, precise localization of the bleeding source was not possible as endoscopic evaluation was not performed.

Patients with hiatal hernia are also at risk of frequent respiratory infections due to increased rate of gastroesophageal reflux‐related micro‐aspirations that are effectively treated with repair of the hernia [16]. However, our patient had only two episodes of pneumonia in the last 2 years.

In conclusion, hiatal hernia should be recognized as a rare but treatable cause of iron refractory IDA, with early diagnosis and surgical repair effectively reversing the anemia and preventing associated complications.

## Author Contributions

**Agete Tadewos Hirigo:** data curation, formal analysis, methodology, resources, validation, writing – original draft, writing – review and editing. **Worku Ketema:** conceptualization, investigation, methodology, resources, validation, visualization, writing – original draft, writing – review and editing. **Kefyalew Taye Garie:** conceptualization, data curation, formal analysis, investigation, methodology, resources, software, supervision, validation, visualization, writing – original draft, writing – review and editing. **Enatnesh Terefe Mame:** conceptualization, data curation, formal analysis, funding acquisition, investigation, methodology, resources, software, supervision, validation, visualization, writing – original draft, writing – review and editing. **Mekdes Shifeta:** conceptualization, formal analysis, investigation, methodology, validation, visualization, writing – original draft, writing – review and editing. **Wondimagegn Gizaw:** conceptualization, data curation, investigation, writing – original draft, writing – review and editing. **Shifte Hamid:** conceptualization, data curation, investigation, resources, validation, visualization, writing – original draft, writing – review and editing. **Rihana Kassim Ahmed:** data curation, investigation, visualization, writing – original draft, writing – review and editing. **Tsegaye Yasin:** data curation, investigation, methodology, software, visualization, writing – original draft, writing – review and editing. **Solomon Kelemu Leykun:** data curation, investigation, validation, visualization, writing – original draft, writing – review and editing. **Anteneh Dejene:** data curation, investigation, methodology, validation, visualization, writing – original draft, writing – review and editing. **Mulugeta Sitot Shibeshi:** investigation, validation, visualization, writing – original draft, writing – review and editing.

## Funding

The authors have nothing to report.

## Ethics Statement

Written informed consent for the publication of this case report was obtained from the child's parents.

## Consent

Written informed consent was obtained from the patient's parent(s) for the publication of anonymized information in this article.

## Conflicts of Interest

The authors declare no conflicts of interest.

## Data Availability

The data that have been used is confidential.
